# Advances in Nrf2 Signaling Pathway by Targeted Nanostructured-Based Drug Delivery Systems

**DOI:** 10.3390/biomedicines12020403

**Published:** 2024-02-09

**Authors:** Sarmistha Saha, Nadezhda Sachivkina, Arfenya Karamyan, Ekaterina Novikova, Tamara Chubenko

**Affiliations:** 1 Department of Biotechnology, Institute of Applied Sciences & Humanities, GLA University, Mathura 281406, India; 2 Department of Microbiology V.S. Kiktenko, Institute of Medicine, Peoples’ Friendship University of Russia (RUDN University), 117198 Moscow, Russia; sachivkina@yandex.ru; 3 Department of Veterinary Medicine, Agrarian Technological Institute, Peoples’ Friendship University of Russia (RUDN University), 117198 Moscow, Russia; arfenya@mail.ru (A.K.); 1032200498@rudn.ru (E.N.); 1032201888@rudn.ru (T.C.)

**Keywords:** nanotechnology, Nrf2 signaling pathway, drug delivery, oxidative stress, drug targeting agents

## Abstract

Nanotechnology has gained significant interest in various applications, including sensors and therapeutic agents for targeted disease sites. Several pathological consequences, including cancer, Alzheimer’s disease, autoimmune diseases, and many others, are mostly driven by inflammation and Nrf2, and its negative regulator, the E3 ligase adaptor Kelch-like ECH-associated protein 1 (Keap1), plays a crucial role in maintaining redox status, the expression of antioxidant genes, and the inflammatory response. Interestingly, tuning the Nrf2/antioxidant response element (ARE) system can affect immune–metabolic mechanisms. Although many phytochemicals and synthetic drugs exhibited potential therapeutic activities, poor aqueous solubility, low bioavailability, poor tissue penetration, and, consequently, poor specific drug targeting, limit their practical use in clinical applications. Also, the therapeutic use of Nrf2 modulators is hampered in clinical applications by the absence of efficient formulation techniques. Therefore, we should explore the engineering of nanotechnology to modulate the inflammatory response via the Nrf2 signaling pathway. This review will initially examine the role of the Nrf2 signaling pathway in inflammation and oxidative stress-related pathologies. Subsequently, we will also review how custom-designed nanoscale materials encapsulating the Nrf2 activators can interact with biological systems and how this interaction can impact the Nrf2 signaling pathway and its potential outcomes, emphasizing inflammation.

## 1. Introduction

Inflammation is an adaptive immune response in response to injury, infection, or other harmful stimuli [1]. Under normal physiological conditions, inflammation leads to the restoration of proper functioning of tissues. However, this could lead to chronic tissue damage. Inflammation is a common phenomenon in a number of pathological conditions, such as tumors [2], sepsis [3], atherosclerosis [4], traumatic brain injury [5], and many more.

Uncontrolled inflammation is a significant issue and is linked with several pathological consequences [6,7]. Therefore, understanding the inflammatory mechanisms can be beneficial to design novel therapeutics for several diseases. It is particularly pertinent that inflammation is linked to redox state changes [8], causing oxidative stress and cellular injury. In this context, the cross-talk between inflammation and oxidative stress is worth a target for drug discovery and poses significant challenges. The production and release of soluble signaling molecules, termed cytokines, characterize the inflammatory response. The pathogen-associated molecular patterns (PAMPs), which uniquely recognize the molecules expressed by the pathogens, are responsible for the initial detection of an infection signal and/or damaged tissues [9]. Endogenous chemicals, known as damage-associated molecular patterns (DAMPs), detect dying cells and trigger the immune system by interacting with pattern recognition receptors (PRRs). Upon successful detection of these signals by transmembrane Toll-like receptors (TLRs), NF-κB (nucleus kappa light chain enhancer of activated B) is activated. As a result, NF-κB translocates to the nucleus and splits from IκB, activating the transcription process, which in turn activates the pro-inflammatory cytokines, such as interleukin-1-beta (IL-1β), IL-6, tumor necrosis factor-alpha (TNF-α), and others, in the subsequent stage of the cascade [10]. Reactive oxygen and nitrogen species (ROS, RNS), which cause damage to macromolecules like proteins and DNA, are produced when these immune cells, such as neutrophils and monocytes, are recruited to the site of the injury. Normal restoration restores tissue homeostasis and prevents further neutrophil recruitment for wound healing. A persistent inflammatory response that results in tissue injury increases the cellular damage in chronic “inflammation”. The transcription factors, including NF-κB and NF-E2-related factor 2 (Nrf2), are the crucial components of inflammation and oxidative stress pathways [11]. Numerous studies have demonstrated that activation of Nrf2-related systems is an effective way to protect from tissue injury in a variety of disease conditions. However, a number of issues, including poor solubility and membrane permeability, limited bioavailability, low stability, short half-life, and rapid clearance, limit the use of Nrf2 modulators in clinical settings [12].

Nano–bio interfaces are where nanoscale surfaces meet biology to control metabolism, the immune system, the cell cycle, and disease-associated pathways. The phrase “nanostructures” refers to a wide range of materials with varied dimensions and forms. The use of therapeutic, imaging, and diagnostic nanostructures composed of superparamagnetic iron oxide, silver, gold, lipids, carbon-based materials, proteins, and polymers is growing. Due to their distinctive properties, such as high aspect ratio, surface area, and targeted and controlled delivery, nanotechnology is a viable platform for the treatment of immune-related disorders as well as the therapeutic and vaccine administrations [13]. However, there are several challenges that still exist. In every scenario, tissue-resident macrophages and circulating monocytes are principally responsible for engulfing nanostructures [14]. It is feasible to modify immune cells in order to circumvent the limiting efficiency of nanosystems because immune cells have the ability to self-regulate. However, to customize nanoparticles to a particular disease condition, the system’s tenability is crucial. Since immune cells have the ability to self-regulate, it is possible to manipulate them in order to circumvent the limiting efficiency of nanosystems.

This article considers the mechanism of how the Nrf2 signaling pathway could be a potential therapeutic target for inflammation and oxidative stress-related diseases. We will discuss how nanomaterial-based drug delivery systems and interactions with biological systems could influence the Nrf2 signaling pathway for their efficient clinical translations.

## 2. Nrf2-Keap1 Signaling Pathway

**Figure 1:**
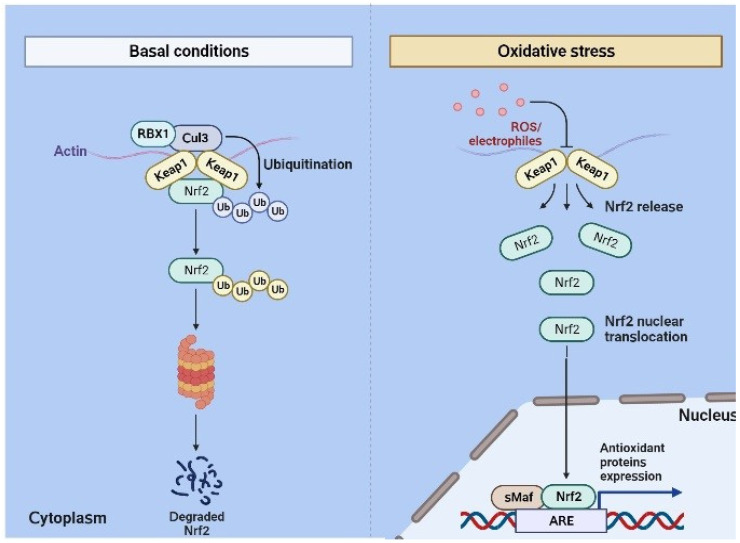
Nrf2 signaling pathway. Keap1 homo-dimerizes via the N-terminal BTB domain under normal homeostatic conditions, and it binds to the (Cul3) E3 ligase to form the Keap1-Cul3-RBX1-E3 ligase complex, which causes Nrf2 ubiquitination. Stress causes Nrf2 to be released from the Keap1-Cul3-RBX1 complex and move into the nucleus, where it binds to the antioxidant response elements (AREs) and hetero-dimerizes with sMaf proteins to cause the transcription of ARE-driven genes.

Nrf2 (NF-E2-related factor 2) comprises 605 amino acids and conserved domains from Neh1 to Neh7. The N-terminal domain is responsible for the stability and ubiquitination of Nrf2 by interacting with Keap1 via binding motifs such as ETGE and DLG [15]. The Neh1 domain is responsible for DNA binding via the basic-region leucine zipper (bZIP) domain [16]. Next, Neh3 to Neh5 domains mediate the interaction of Nrf2 with other coactivators [17,18]. Another domain, Neh6, is responsible for ubiquitination via ß-transducin repeat-containing protein [19]. Finally, Neh7 inhibits the ARE signaling pathway via the retinoic X receptor. Keap1 is a cysteine-rich protein with 624 amino acids [20]. The BTB domain in Keap1 leads to the homo-dimerization for binding to the E3 ligase, leading to the formation of the Keap1-Cul3-RBX1 (Ring box protein-1) E3 ligase complex [21]. Similarly, the Kelch repeats lead to the binding of Keap1 to Nrf2 and p62 [22,23]. Oxidative stress and electrophile attack could lead to the uncoupling of the DLG motif from Keap1, releasing Nrf2 from the Keap1-Cul3-RBX1 complex. This sequence is followed by the translocation of Nrf2 into the nucleus, leading to the hetero-dimerization with small Maf proteins (sMaf) and further binding to the antioxidant response element (ARE) (Figure 1). This further activates several antioxidant enzymes, such as heme oxygenase-1 (HO-1) and glutathione-S-transferase (GST) [24]. It is now well recognized that thiol modifications of C151 in the BTB domain inhibit the Keap1-Cul3 interactions and finally activate Nrf2 [25].

The rate-limiting step in oxidative heme breakdown is catalyzed by the inducible 32 kDa protein known as heme oxygenase-1 (HO-1, HMOX1, EC 1.14.99.3). Heme is transformed during this process into three bioactive molecules, free iron, carbon monoxide (CO), and biliverdin, which is quickly converted to bilirubin and is essential for inflammation, apoptosis, and oxidative stress [26]. Similar to other antioxidant proteins, Nrf2 directly regulates the expression of the HO-1 enzyme-coding gene, HMOX1. A number of in vitro and in vivo studies were conducted to support the crucial role that Nrf2 mediates for the production of HO-1 in anti-inflammatory activity. In a study on macrophage polarization, the expression of CD163 and HO-1 identified in vitro M-CSF-polarized macrophages. Subsequent research revealed that treatment with cobalt protoporphyrin, which triggers HO-1 activity, increased the release of IL-10 in M2 macrophages. This suggests that the CD136/HO-1/IL-10 axis can influence the anti-inflammatory properties of M2 macrophages. Similar to this, HO-1 activation in macrophages via hemin was a factor in the anti-inflammatory effect in acute pancreatitis [27]. Parkinson’s disease was markedly worsened by the expression of COX-2, iNOS, IL-6, and TNF-α after Nrf2 was silenced by a knockout [28]. The increased production of inflammatory proteins (COX-2 and iNOS) and inflammatory cytokines (TNF-α and IL-6) was discovered to be followed by an increase in HO-1 and NADPH quinone oxidoreductase I (NQO1) levels, indicating the involvement of the Nrf2/HO-1 axis in inflammation. Numerous oxidative/electrophilic stresses have the ability to rapidly induce HO-1, which can then prevent immune cells from producing ROS and NO [29].

The production of prostaglandins, thromboxanes, and levuloglandins by inflammatory enzymes known as cyclooxygenases (COX) is also necessary for the inflammatory response [30]. When it comes to acute inflammation, COX-2 is the most important COX enzyme [31]. In the beginning, macrophages are the main cells that express COX-2, and they also activate it in response to growth factors. Nrf2-deletion mice produce significantly more proinflammatory cytokines than their wild-type counterparts, suggesting a potential function for Nrf2 in suppressing COX signaling [32]. Similarly, 1-methyl-4-phenyl-1,2,3,6-tetrahydropyridine stimulation resulted in a considerable rise in oxidative stress and the production of COX-2, iNOS, IL-6, and TNF-α in Nrf2-knockout animals [28]. Pretreatment with Nrf2 activators, such as sulforaphane, can reduce proinflammatory cytokines in peritoneal macrophages, which can have anti-inflammatory effects; however, Nrf2-knockout mice did not exhibit this effect [33].

Numerous research studies looked into probable mechanisms underlying the control of the NLRP3 inflammasome by Nrf2. It is thought that Nrf2 inhibits the activation of NLRP3 via reducing ROS generation because it regulates the expression of antioxidant genes and oxidative stress [34]. Furthermore, Nrf2 reduces the expression of genes, including NLRP3, caspase 1, IL-1β, and IL-18, that are involved in inflammasome assembly, hence preventing the activity of the NLRP3 inflammasome [35]. Importantly, it was shown that tert-butylhydroquinone (tBHQ), a known Nrf2 activator, increased the expression of NQO1 and inhibited NLRP3 priming in a way that was Nrf2–ARE dependent [36]. These findings point to Nrf2 as a potential new therapeutic target for NLRP3 inflammasome complex assembly inhibition.

**Figure 2:**
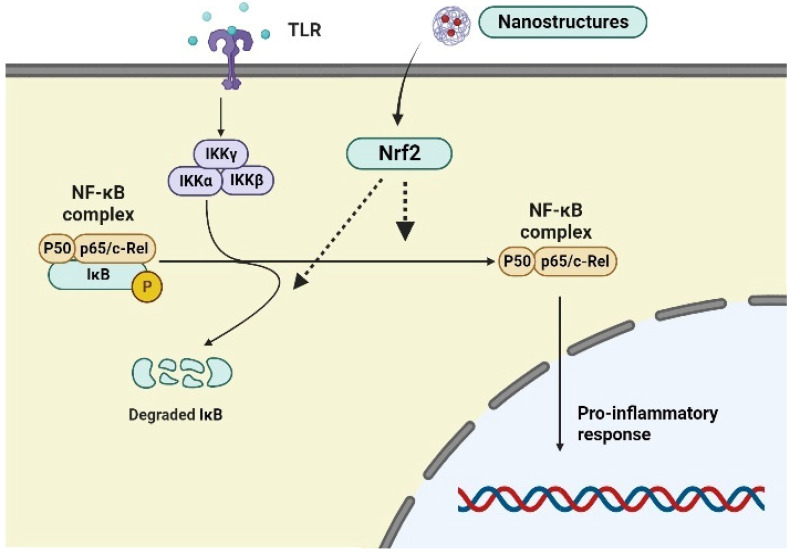
Cross-link of Nrf2 and NF-κB in inflammation. The IKK complex phosphorylates NF-κB, freeing it from IκB in response to TLR signaling. Following its translocation into the nucleus, NF-κB stimulates the production of proinflammatory mediators. Nanostructures stimulate Nrf2, which prevents IκB-α degradation and oxidative stress-mediated NF-κB activation and nuclear translocation.

The Nrf2 and NF-κB signaling pathways coordinate with each other for the regulation of redox imbalance and inflammation. NF-κB constitutes a complex system of transcription factors for regulating innate and adaptive immunity, inflammatory responses, and oxidative stress. In the presence of harmful stimuli, the canonical NF-κB is activated and released from the IκB kinase complex due to the phosphorylation of the IKK complex, ultimately leading to their translocation into the nucleus and release of the pro-inflammatory modulators (Figure 2). Activation of the IκB kinase complex and subsequent phosphorylation of IκB proteins cause their ubiquitination and proteasomal degradation when specific stimuli such as proinflammatory cytokines and oxidative stress are present. Consequently, p50/RelA and p50/c-RelNF-B dimers are unrestricted in their ability to translocate into the nucleus and activate a variety of target genes. The transcription factor NF-κB, which controls inflammatory reactions and cellular damage, is likewise redox-regulated [37]. Following nuclear translocation, NF-B stimulates the expression of inflammatory cytokines, COX-2, iNOS, vascular adhesion molecules, and other inflammatory molecules [37]. From a functional standpoint, Nrf2 exerts a variety of negative restrictions on the NF-κB signaling pathway. Nrf2 inhibits NF-κB activation brought on by oxidative stress by lowering intracellular ROS levels [38]. Additionally, Nrf2 suppresses NF-κB’s nuclear translocation and IκB-α proteasomal degradation [39]. Increased production of phase II enzymes prevents IκB-α degradation by increasing cellular HO-1 levels, which are caused by the upregulation of Nrf2 [40]. Increasing data also indicate that Nrf2 competes with transcription co-activator cAMP response element (CREB) binding protein (CBP) to inhibit the NF-B-driven inflammatory response [41]. It is interesting to note that some Nrf2 target genes, including NQO1, glutamate-cysteine ligase catalytic subunit (GCLC), and glutamate-cysteine ligase modifier subunit (GCLM), may also have NF-κB binding sites. This may be the cause of drugs that target NF-κB signaling activating the Nrf2 pathway instead [42]. It is now well recognized that the Nrf2 signaling pathway suppresses oxidative stress-induced activation of NF-κB and IκB-a proteasomal degradation [43]. Additionally, recent research has shown that, through the activation of the protein Rac1, NF-κB induction can increase Nrf2 activity [44]. Furthermore, the inhibition of NF-κB dependent inflammatory activity by the antioxidant enzyme HO-1 was found, indicating the critical role of Nrf2 in reducing oxidative stress [44].

**Figure 3:**
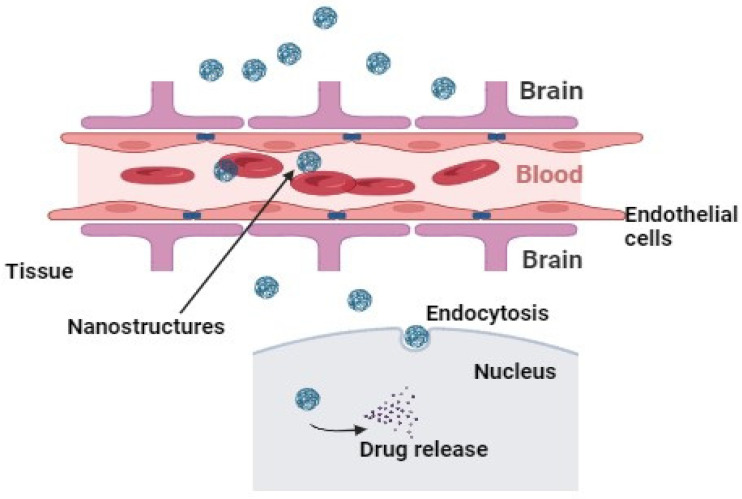
The passive drug targeting in tissue penetration from nanostructures. The design and synthesis of nanostructures are composed in such a way that their retention time in the body increases, correlated with particle size.

Although potent anti-inflammatory agents, such as corticosteroids, adenosine, NSAIDs, and many others, have shown promising results against inflammation, they have shown negative effects on tissue repair, rapid clearance, and unacceptable toxic effects [45]. Another challenge is the initial innate responses that disturb different signaling pathways, causing cellular deterioration and organ failure. Currently, dimethyl fumarate (DMF) is the only drug approved by US Food and Drug Administration and marketed by Biogen to treat multiple sclerosis by targeting the Nrf2 signaling pathway [46,47]. Some other prodrugs of monomethyl fumarate are currently undergoing phase III clinical trials for multiple sclerosis patients. Similarly, tepilamide fumarate is undergoing phase II clinical trials for plaque psoriasis treatment [48]. Emerging evidence also reveals that some dietary polyphenols could induce Nrf2 for the treatment of oxidative stress and inflammation [49]. Similarly, the supplementation of antioxidants has also been studied [50] to scavenge oxidative stress and simultaneous inflammation. However, limited pharmacokinetics and cellular penetration make them unable to treat pathological redox imbalances inside cells and tissues [51]. Another major challenge includes the transport of drug molecules through biological barriers. Moreover, efficient therapeutic delivery agents are also needed at the targeted inflammation site against such processes. Nanotechnology-based drug delivery systems could control particle size, shape, surface charge, and drug loading for specific applications. The two major targeting strategies for drug targeting include active targeting through site-specific ligands and passive targeting via increased permeability and retention effects (Figure 3). To improve these therapeutic issues, in the next section, we will discuss potential multidrug nanostructures that target the Nrf2 signaling pathway for the resolution of inflammation.

## 3. Nanotechnology-Based Targeting of Inflammation via the Nrf2 Signaling Pathway

Nrf2 activation has been linked to inflammation and reportedly hinders the transcription of pro-inflammatory cytokines, including IL-1β and Il-6 [52]. Also, Nrf2 activators, such as tBHQ [53], Ebselen [54], and CDDO-Me analog dh404 [55], augment endogenous antioxidants and counteract the inflammatory response to prevent disease progression.

### 3.1. Organic Nanostructures

Sabzichi et al. [56] formulated luteolin in nanophytosomes and tested their efficacy in MDA-MB 231 cells. They observed the suppression of Nrf2 and downstream genes like HO-1 and MDR1. These changes collectively led to significantly high apoptosis of cancer cells compared with luteolin alone. In earlier studies, luteolin proved to be an inhibitor of Nrf2; however, poor solubility and penetration restricted its application, which was overcome in this study by using nano-based phytosomes [57].

Yan et al. [58] reported a nanospray of ligustrazine on postoperative abdominal adhesion in rats, and it was observed that protein expressions of MMP-9, Nrf2, heme-oxygenase-1, and NQO1 were increased. In the same study, MCP-1 protein, TNF-α, and IL-1β were also found to be elevated. Similarly, a nanoformulation of roselle seed oil significantly inhibited the paracetamol-induced mRNA expression of pro-inflammatory cytokines, including TNF-α and IL-6 [59]. The same study also showed increased levels of Nrf2 and glutathione due to nanoformulation treatment. Pandhita et al. [60] reported a nano-based formulation of curcumin against cisplatin-induced renal injury. They observed upregulation of Keap1 and Nrf2 activation due to nanoformulation treatment. In addition, chitosan-coated curcumin nanocrystals were investigated against an LPS-induced sepsis model and HepG2 and J774 cells [61]. The nanocrystals conferred protection via Nrf2 activation with elevated SOD and GST levels. The results also revealed NF-κB downregulation and suppressed cytokine levels. Likewise, another study also showed that the liposomal nanocurcumin alleviated copper sulfate (CuSO_4_)-induced testicular lipid peroxidation, inflammation, and apoptosis via Nrf2/HO-1 signaling [62]. Here, it was postulated that the nanocurcumin activates Nrf2/HO-1 signaling by promoting the levels of Nrf2 and Bcl-2 with the enhancement of endogenous antioxidants, such as HO-1, GSH, and SOD, thereby protecting from tissue injury.

One of the most potent Nrf2 activators is CDDO-Me, a triterpenoid oleanolic acid analog that reportedly regulates inflammation, oxidative stress, and cell death. CDDO-Me is undergoing a Phase I trial in cancer patients [63]. To this end, in a most recent study, encapsulation of an Nrf2 activator, CDDO-methyl into polymeric nanoparticles, led to the upregulation of the expression of antioxidants and cytoprotective proteins for athero-protection [64]. They showed the accumulation of nanoparticles in atherosclerotic plaque of ApoE^−/−^ and LDLr^−/−^ mice, which were accompanied by the activation of Nrf2. The targeted delivery of nanoparticles to atherosclerotic plaque led to the elevation in expression of the Nrf2-regulated genes *GCLC* and *NQO1*. This study supports the paradigm that nanosystem administration of redox-active therapeutics can counteract the inflammatory response via Nrf2 activation. Furthermore, in another study, a molecular probe biosensor based on a multiplexed microfluidic device was designed as a nanoengineered platform to identify potent Nrf2 activators [65]. Similarly, nanoselenium is a potent antioxidant [66] and, therefore, has been studied for its antioxidant activity against cadmium-induced hepatotoxicity. The results revealed that nanoselenium mitigates oxidative stress via the Nrf2 signaling pathway and up-regulates the expressions of Nrf2 and their downstream targets, including HO-1, NQO-1, GST, and SOD [67].

Oridonin nanoparticle was developed using polyethylene glycol and poly-lactic-co-glycolic acid and proved effective against breast cancer by inducing ROS generation and also activating the Nrf2/HO-1 signaling pathway [68]. The polyethylene glycol-capped gold nanoparticles prevented acute kidney injury caused by renal ischemia-reperfusion by lowering lipid peroxidation, IL-1β, and TNF-α; upregulating the AMPK/PI3K/AKT pathway; and increasing Nrf2 expression [69].

The lipid-based nanotechnology for drug delivery systems can be applied in many forms, and solid lipid nanoparticles are the most effective and stable form. Interestingly, solid lipid nanoparticles exhibited several unique advantages, such as tissue targeting, controlled drug release kinetics, minimal immune response, high bioavailability, and loading efficiency [70]. Some reports stated that the encapsulation of oridonin, a natural product with solid lipid nanoparticles, enhanced the antitumor effect in different cancer cell lines, such as breast cancer and hepatocellular and lung cancer cells [71]. Here, nanoparticles induced cell cycle arrest at S and apoptotic rates. In this regard, another investigation demonstrated the importance of the Nrf2–NF-κB cross-link in anticancer activity [72]. When astaxanthin was encapsulated into solid lipid nanoparticles, a protective effect was observed against DMBA-induced breast cancer via the mTOR/Maf-1/PTEN pathway [73]. Moreover, astaxanthin-loaded nanoparticles suppressed p-AKT levels, further suppressing the expression of NF-κB and Keap1 with a simultaneous elevation of HO-1 and Nrf2 expressions. In agreement with these results, the antioxidant, anti-inflammatory, and neuroprotective activity of resveratrol-encapsulated solid lipid nanoparticles significantly improved the redox state and suppressed ROS generation [74]. The nanoparticles also caused a reduction in hypoxia-inducible factor 1α (HIF-1α) levels with Nrf2 activation and promoted the expression of HO-1 in vascular dementia [74]. The nanoparticle treatment caused a significant elevation in Nrf2 mRNA expression in the cortex, hippocampus, striatum, and cerebellum. Moreover, another report identified copolymers and trigonelline-entrapped micelles for colon cancer treatment [75]. These micelles had spherical shapes and significantly inhibited Nrf2 activation and ARE-related gene expressions. Furthermore, trigonelline-entrapped micelles enhanced oxaliplatin-induced apoptosis in an Nrf2-dependent manner. Emerging evidence also reveals that apigenin–solid lipid nanoparticles modulate oxidative stress and inflammation in diabetic nephropathy [76]. The anti-inflammatory effect of the nanosystem was studied in the mRNA expression of cytokines, such as IL-6, TNFα, and IL-1β. Moreover, treatment with nanoparticles promoted Nrf2 and HO-1 expression with simultaneous suppression of NF-κB activity and lipid peroxidation, suggesting the existence of antioxidant and anti-inflammatory properties.

Shahin et al. [77] designed, synthesized, and evaluated the potential of caffeic acid phenethyl ester-loaded nanoliposomes in a rat model of acute pancreatitis. They observed protection against malondialdehyde, NF-κB, TNFα, and caspase-3 activity. Furthermore, nanoliposomes also caused Nrf2 activation and up-regulated gene expressions of antioxidants such as glutathione reductase and HO-1, leading to modulation in the Bcl-2/Bax ratio. Thus, the nanoliposomal system more profoundly counteracts oxidative stress and inflammatory response by targeting Nrf2 activation compared with standard drugs without a nanosystem due to efficient cellular uptake and improved pharmacokinetics. A study found that long non-coding RNA (MT1DP) could effectively sensitize A549 and H1299 cells by down-regulating Nrf2 through stabilizing miR-365a-3p and up-regulating ROS, which in turn causes erastin-induced ferroptosis in non-small cell lung cancer. This was achieved by developing a nano-delivery system using folate-modified liposomes [78]. With a mean particle size of 80 ± 5 nm, quinacrine-loaded liposomes, an Nrf2 inhibitor, demonstrated decreased Nrf2 expression. This was followed by its downstream genes, MRP1, Trx, and bcl2, which ultimately produced A549 lung cancer cells that were more sensitive to cisplatin [79].

### 3.2. Inorganic Nanostructures

Graphene oxide (GO) is a two-dimensional nanomaterial, and its particle-like properties make them suitable for studies with inflammatory responses. The effects of different GO sheets (small, ultrasmall, and large) were investigated on inflammatory responses in macrophages [80]. They found that GO small sheets inhibit IL-1β, IL-6, and TNF-α secretion. Specifically, the authors suggested that the GO small sheets could not influence NLRP3 inflammasome activation; however, they reduced the expression of pro-IL-1β. It was suggested that the GO small sheets are efficiently internalized and inhibit the expression of IL-1β and IL-6 mRNA in LPS-stimulated macrophages. To this end, the authors then investigated the mechanism of this inflammatory gene regulation, and they found that Nrf2 activation up-regulates the expression of anti-inflammatory genes and suppresses IκBζ−NF-κB-mediated pro-inflammatory mediators, including IL-1β and IL-6.

Among several nanoparticles, silica nanoparticles have been shown to induce an inflammatory response even after short-term exposure, owing to their large surface area and optical transparency. Nano-SiO_2_ has been reported to induce carcinogenesis via suppressed DNA methylation [81]. In cancers, Nrf2 plays a contrary role, and its loss leads to malignant cellular transformation [82]. In contrast, accumulating evidence also suggests that Nrf2 activation promotes cancer cell growth and metastasis in different tumor types [83,84,85]. In this context, nano-SiO_2_ exposure leads to carcinogenesis in human bronchial epithelial cells via DNA hypomethylation and altered methylCpG binding protein expression. They also observed Nrf2 activation by CpG island demethylation within the promoter region of Nrf2. All these changes lead to the Nrf2 upregulation, which might be the reason for alleviating the nano-SiO_2_-induced carcinogenesis [86]. Furthermore, they reported an enhancement in antioxidants, such as HO-1, SOD1, and GST levels, due to nano-SiO_2_ exposure. In an earlier study, it was reported that nano-SiO_2_ (10–20 nm) exposure up-regulated the levels of PKR-like endoplasmic reticulum (ER)-regulated kinase (PERK), Nrf2, and HO-1 in A549 cells [84]. Next, they developed A549-shNrf2 cells and observed Nrf2 activation due to nano-SiO_2_ exposure; however, the changes observed were less profound than the A549 cells. It was also reported that the Nrf2 expression level was reduced significantly in Nrf2^−/−^ ICR mice exposed to nano-SiO_2_ along with ROS elevation compared to the wild type. Thus, these results suggest that Nrf2 activation protects against nano-SiO_2_-induced toxicity. In a recent study, Argenziano and co-authors [87] designed a chitosan-shelled nanobubble (siNrf2-NBs) for siRNA against Nrf2, demonstrating the downregulation of target genes in M14 cells. The treatment was administered in combination with ultrasound to treat melanoma cancer cells.

Nowadays, gold nanoparticles (AuNPs) and their derivatives have also been a focus for their different applications [88]. However, AuNPs also reportedly show toxicity signs [89,90,91]. In this context, Bajak and co-workers studied the plasmonic excitation effects of AuNPs (5 and 30 nm) on Caco-2 cancer cells [92]. Interestingly, small AuNPs (5 nm) induced the expression of Nrf2-responsive genes, such as *HMOX*, *G6PD,* oxidative stress-induced growth inhibitor 1 (*OSGIN1*), and glutathione peroxidase 2 (*GPX2*). Furthermore, these results revealed oxidative stress and a disturbed redox state due to the AuNPs, indicating their anticancer property via the Nrf2 pathway. However, low concentrations of AuNPs affect GSH levels only slightly and, thus, activate Nrf2 in a ROS-dependent manner. Here, it is worth mentioning that γ-glutamyl cysteine ligase and glutathione synthetase, which are associated with GSH synthesis, are regulated by the Nrf2 pathway [93]. Thus, the interaction of AuNPs to GSH could be related to Nrf2 activation. Nrf2 activation by AuNPs and their plasmonic excitation could be a new platform for treating several pathologies. In a murine model of streptozotocin-induced diabetic nephropathy, pomegranate peel extract was tested in conjunction with stabilized gold nanoparticles. It reduced inflammatory mediators by modulating the MAPK/NF-κB/STAT3/cytokine axis and also activated PI3K/AKT/Nrf2. Eventually, this led to an increase in antioxidant enzymes, a decrease in blood glucose, a decrease in triglycerides, an increase in insulin, and a return of pancreatic β-cell dysfunction [94].

In another study, both silver (AgNPs) and AuNPs reportedly showed uptake into the cytoplasm; however, no uptake was observed within the nucleus of Caco-2 cells [95]. Also, the AgNPs reduced the total glutathione and upregulated the expression of Nrf2 and HO-1. However, these AgNP-induced changes were significantly reversed by siRNA silencing of Nrf2 transcripts [95]. A study in Nrf2-knockout HK-2 renal epithelial cells treated with AgNPs showed G2/M growth arrest, high phospho-CDC25C, phospho-CDC2, and GSH depletion [96]. In addition, it was observed that the pre-treatment with N-acetylcystein caused Nrf2 pathway upregulation of GCLC and GCLM expression, thereby enhancing the GSH levels. Therefore, taken together, all these results show that the Nrf2–GSH pathway could be involved in the AgNP-mediated toxicity in epithelial cells.

8-Oxoguanine DNA glycosylase 1 (OGG1) is a DNA repair protein marker for ROS generation. When human Chang hepatocytes were treated with AgNPs, OGG1 protein expression was significantly suppressed [97]. They revealed that AgNPs downregulated OGG1 expression with concealed Nrf2 binding to the promoter region of OGG1 and, thereby, decreased nuclear Nrf2 levels. It has also been reported that an Nrf2 transcription factor binding site is localized in the promoter region of the human OGG1 region, which correlates the levels of OGG1 with the redox state of the cells [98]. ERK and AKT reportedly phosphorylate Nrf2, leading to the Nrf2 release from Keap1, followed by its translocation into the nucleus [99,100]. The results revealed that AgNPs downregulate ERK and AKT and, further, OGG1, intensifying oxidized DNA bases. Nrf2 and stimulation protein-1 (SP-1) have binding sites in the OGG1 promoter region [98]. On the other hand, the AKT pathway plays a crucial role in regulating Nrf2 and SP-1 [101,102]. Similarly, Nrf2 activation is also regulated by the ERK pathway and the upregulation of OGG1 [103,104]. Therefore, the AKT and ERK pathways are responsible for the upregulation of OGG1.

It is also now established that iron oxide nanoparticles loaded with curcumin (FeNPs) exhibit ROS-scavenging ability in chondrocytes and reduce the elevated levels of IL-1β [105]. These results were further corroborated by the observation that Nrf2 activation occurred along with the suppression of the nod-like receptor protein-3 inflammasome (NLRP3) activation. Also, all these changes lead to the inhibition of matrix-degrading proteases and other inflammatory factors. Quite recent data report that bovine serum albumin-encapsulated magnetite nanoparticles entrapped with epigallocatechin 3-gallate induced ROS generation and apoptosis in lung adenocarcinoma cells [75]. The nanoparticles exhibited elevated Nrf2 and Keap1 expressions, elevating cell apoptosis.

Similarly, studies on cobalt nanoparticles (CoNPs, 30 nm), mostly used in artificial joint replacements, showed ROS generation and activation of the Nrf2 signaling pathway [106]. On the other hand, selenium nanoparticles (SeNPs) suppress oxidative stress in cells [107,108]. Multiple studies studied the potential mechanisms underlying the antioxidant activity of SeNPs in different cell lines. In IPEC-J2 cells, SeNP treatment activated Nrf2, mitogen-activated protein kinase (MAPK), and the AKT pathway and simultaneously raised the levels of HO-1 and NQO-1 [109]. Moreover, SeNP treatment also promoted phosphorylated-Nrf2 levels without affecting Keap1. Importantly, this phosphorylation was reportedly mediated via p38, ERK1/2, c-Jun N-terminal kinase, and the AKT pathway. Similarly, biogenic SeNPs synthesized using bacteria showed maintenance of intestinal redox homeostasis via an Nrf2-mediated pathway [110]. Biogenic SeNPs (Nrf2) activated Nrf2 and upregulated the expressions of thioredoxin reductase (TXNRD)-1, NQO-1, HO-1, and thioredoxin (Trx) in concentration-dependent manners. These results were more significant as compared with chemically synthesized SeNPs.

In cancer treatment, photothermal therapy and photodynamic therapy proved highly beneficial due to their target selectivity, non-invasive nature, and negligible drug resistance [111,112]. However, the intracellular antioxidant system will partly hinder phototherapy for anticancer treatments. In some instances, the Nrf2 signaling pathway has been shown to promote the resistance of tumor cells to phototherapy [113]. Thus, Nrf2-specific siRNA could be a potential photosensitization strategy to promote phototherapy efficiency via the Nrf2 pathway [114]. However, the targeted delivery of Nrf2–siRNA in tumor cells is also necessary [115,116]. In this regard, a potential nanosystem, including poly(β-amino ester)/poly lactic-co-glycolic acid-based nanoparticles encapsulated with indocyanine green and Nrf2–siRNA, was designed and developed [117]. This nanosystem was then further loaded inside the vesicles of cell membranes derived from oral tongue squamous cell carcinoma cells. These nanoparticles suppressed the Nrf2 signaling pathway and promoted anticancer phototherapy by enhancing ROS accumulation. All these changes led to the apoptosis of cancer cells and reduced tumor growth in animal models. In another report, lung cancer cell death occurred via ferroptosis as a result of zero-valent-iron nanoparticle (ZVI-NP)-induced mitochondrial malfunction, intracellular oxidative stress, and lipid peroxidation. Such cancer-specific ferroptosis increased the degradation of Nrf2 by GSK3/-TrCP via activation of AMPK/mTOR [118]. The authors also revealed the tuning of macrophage polarization from the immunosuppressive M2 phenotype to the antitumor M1 phenotype and suppression of PD-1 and CTLA4 in CD8+ T cells, thereby inducing ferroptosis.

Titanium dioxide nanoparticles enhanced Nrf2 expression in liver cancer cells along with its associated target genes NQO1, HO-1, and GCLC, while Nrf2 loss in Nrf2(−/−) cells significantly increased susceptibility to DNA damage [119]. After conjugating with spironolactone, zinc oxide nanoparticles were created, which ultimately reduced kidney injury by increasing Nrf2 and HO-1 expressions and decreasing inflammatory mediators like TGF-β1, Wnt7a, β-catenin, fibronectin, and collagen [120]. In streptozotocin-induced diabetic rats, cerium oxide nanoparticles (30 mg/kg bodyweight/day for 4 weeks) enhanced sperm fertility by promoting Nrf2 expression, which inhibited sperm DNA fragmentation [121]. Similarly, in streptozotocin-induced type 1 diabetic Swiss mice, nanoceria effectively decreased glucose levels, lowered IL-6 and TNF-α, increased Nrf2 expression, increased superoxide dismutase, and decreased apoptosis [122].

Zinc oxide nanoparticles disrupted the ubiquitin–proteasome system, effectively stimulating the Nrf2 signaling pathway and reducing endothelial damage [123]. Zinc oxide nanoparticles improved the ability of Nrf2–DNA binding, reduced AGE, inhibited NLRP3-mediated inflammasome activation, reduced interleukins, and triggered Nrf2/TXNIP/NLRP3 inflammasome signaling [124]. In diabetic rats, *Cyperus rotundus*-loaded zinc oxide nanoparticles significantly enhanced antioxidant enzymes and downregulated procaspase-1, caspase-1, IL-18, and IL-1β [123]. In rats exposed to adenine-induced nephrotoxicity, TGF-β1, Wnt7a, β-catenin, fibronectin, collagen IV, α-SMA, TNF-α, and IL-6 were reduced by zinc oxide nanoparticles and the spironolactone-upregulated Nrf2/HO-1 pathway [120]. Similarly, by improving Nrf2 activation and interfering with ubiquitin–proteasome-dependent Nrf2 degradation, copper oxide nanoparticles decreased vascular injury and disease [125].

## 4. Challenges for Clinical Translation

As we discussed, several nano-based drug delivery systems have recently been synthesized using cutting-edge technologies for more practical, regulated, and targeted distribution. However, there are many restrictions and challenges that affect the therapy outcomes that these systems may produce. There is still a long way to go before products based on nanosized therapies for the treatment of inflammation-related complications are approved for clinical use or become commercially available, despite the encouraging outcomes of various nanoparticle-based drug delivery systems based on the Nrf2 signaling pathway. The potential of the majority of these systems has been shown in vitro or in vivo using mouse models; however, there are relatively few human trials, and the outcomes are considerably different from those obtained using animal models. All delivery technologies struggle with target-specific delivery complications. Although it has been discovered that target-specific delivery reduces toxicity and results in more effective treatment, its efficiency cannot be guaranteed until it is able to reach the targeted region in adequate quantities. For instance, when siRNA is provided systemically, it rarely reaches its intended cell or organ because it is rapidly broken down by bodily enzymes and, when given in large doses, its negative charge prevents cells from absorbing it [114]. Micelles and liposomes, which are lipid nanoparticles, are being studied for target drug delivery, but this comes with the drawback that the body’s reactions to these nanoparticles, such as phagocytic absorption and hepatic filtration, lower their effectiveness and may even cause toxicity [115]. Another significant issue facing drug delivery systems in general is the toxicity of the particles employed in distribution; some of the nanomaterials used can be hazardous to human health as well as the environment [116]. These insufficient clinical translations can be attributed to a costly and time-consuming process, scaling difficulties, and biocompatibility and safety concerns. Therefore, a greater knowledge of the interplay between various aspects linked to nanoparticle shape may enable the development of more focused and effective drug delivery systems for more effective therapeutic drug delivery. Moreover, nanotechnology faces significant difficulties that extend outside the realm of science and include issues with regulation, ethics, patenting, and marketing tactics. The patenting regulations need to be changed immediately so that claims can only be made in relation to research findings and not a broad variety of applications. Therefore, we promote future drug delivery systems that employ materials that more precisely and effectively target a particular biological process, are programmable and responsive to biological cues, and yet can be easily integrated to facilitate clinical translation are also something we foresee.

## 5. Conclusions

Combining nanotechnology with immunotherapy could produce a new way to improve the efficacy of immunotherapeutic and nanostructure-based therapy. The Nrf2–Keap1 pathway regulates redox signaling, inflammation, and cellular metabolism. Although much research focuses on Keap1, reaching the target tissue for these compounds and their activity in complex multifactorial diseases still need to be completed. With the ability to specifically target and deliver multiple drugs at tissue foci of inflammation and their ability to scavenge intracellular reactive species simultaneously, nanostructures represent a promising platform to overcome the limitations of both conventional immunotherapeutic and antioxidant therapies. Here, we reviewed ongoing scientific research about nanostructures targeting the Nrf2 signaling pathway to refine our understanding of designing novel drugs. Additionally, this can open the door for endless nanosystem opportunities capable of a more specific and controlled treatment of pathological conditions. However, by a variety of experimental analyses, the relationship between nanoparticles and Nrf2 in humans needs to be studied. The safety and biocompatibility of nanoparticles must be evaluated in a variety of in vitro and ex vivo animal models prior to in vivo preclinical analysis. Therefore, more thorough research is required prior to starting clinical trials, and its safety evaluation must be confirmed before going on sale as a pharmaceutical. Since human results have not yet been extrapolated, it is necessary to evaluate nanostructure safety for therapeutic use and determine how well it modulates Nrf2 in clinical trials. Despite all of the obstacles, we believe that more and more nanoparticles with fewer side effects will be discovered in the near future (Table 1).

## Figures and Tables

**Table 1:** Studies addressing nanostructure-based drug delivery systems loaded with Nrf2 modulators.

Drug Delivery System	Encapsulated Drug	Size (nm)	In Vitro/In Vivo Results	Ref.
Polylactic acid-based nanospray	Ligustrazine	210 ± 0.96 nm	Pharmacological studies showed reduced postoperative abdominal adhesions by the activation of the Nrf2 signaling pathway with reduced oxidative stress and inflammatory response.	[59]
Chitosan-coated nanoparticles	Curcumin	232.1 ± 8.46 nm	Animal studies showed protection against copper sulfate (CuSO_4_)-induced testicular lipid peroxidation, inflammation, and apoptosis via Nrf2/HO-1 signaling.	[62]
Liposomes	Caffeic acid phenethyl ester	476 ± 9.00 nm	Oral pretreatment significantly counteracted ornithine-induced oxidative stress, inflammation, and apoptotic effects.	[64]
Synperonic-PE-P84 pluronic triblock co-polymer nanoparticles	CDDO-Methyl	234 ± 10.00 nm	In vitro studies showed anti-inflammatory activity in classically activated macrophages. In vivo results showed activation of Nrf2-transcriptional targets in the atherosclerotic aortic arch in ApoE^−/−^ andLDLr^−/−^ mice.	[66]
Chitosan-shelled nanobubble	siRNA against Nrf2	100.2 ± 3.1 nm	Nanobubbles inhibited Nrf2 protein expression in melanoma cells.	[76]
Iron nanoparticles	Curcumin	-	Animal model studies showed Nrf2 activation and NLRP3 inhibition in osteoarthritis model.	[93]
PLA-PCL-PEG-PCL-PLA copolymers	Trigonelline	-	In vitro studies showed increased oxaliplatin-induced apoptosis in a Nrf2/ARE-dependent manner.	[94]
Solid lipid nanoparticles	Resveratrol	286 ± 1.47 nm	In permanent bilateral common carotid artery occlusion (BCCAO)-induced model of vascular dementia in rats, reduced oxidative stress and Nrf2 activation were observed after pretreatment with nanoparticles.	[112]
Stealth lipid nanoparticles	Astaxanthin	130.8 ± 0.2 nm	In vitro studies in cancer cell lines showed suppression of NF-κB and Keap1 with enhancement of HO-1 and Nrf2 expressions.	[111]
PLGA nanocapsules	Epigallocatechin-3-gallate	61.37 ± 5.90 nm	Pharmacological studies showed inhibition of the NLRP-3/caspase-1/IL/1β inflammasome pathway in cisplatin-induced nephrotoxicity	[117]
Zerovalent iron nanoparticles	-	70.17 ± 14.4 nm	Studies in lung cancer cell lines showed enhanced GSK3β/β-TrCP-dependent degradation of NRF2 through activation of the AMPK/mTOR signaling pathway.	[107]

## Data Availability

Not applicable.
